# Potential anti-tumor effects of regulatory T cells in the tumor microenvironment: a review

**DOI:** 10.1186/s12967-024-05104-y

**Published:** 2024-03-20

**Authors:** Yu Li, Cangang Zhang, Aimin Jiang, Anqi Lin, Zaoqu Liu, Xiangshu Cheng, Wanting Wang, Quan Cheng, Jian Zhang, Ting Wei, Peng Luo

**Affiliations:** 1 The Department of Oncology, Zhujiang Hospital, Southern Medical University, Guangzhou, China; 2 Department of Pathogenic Microbiology and Immunology, School of Basic Medical Sciences, Xi’an Jiaotong University, Xi’an, 710061 Shaanxi China; 3 Department of Urology, Changhai Hospital, Naval Medical University (Second Military Medical University), Shanghai, China; 4 Key Laboratory of Proteomics, Beijing Proteome Research Center, National Center for Protein Sciences (Beijing), Beijing, China; 5 Key Laboratory of Medical Molecular Biology, Institute of Basic Medical Sciences, Chinese Academy of Medical Sciences, Department of Pathophysiology, Peking Union Medical College, Beijing, 100730 China; 6 College of Bioinformatics Science and Technology, Harbin Medical University, 157 Baojian Road. Nangang District, Harbin, Heilongiiang, China; 7 Institute of Molecular and Translational Medicine, and Department of Biochemistry and Molecular Biology, Xi’an Jiaotong University Health Science Center, Xi’an, 710061 Shaanxi China; 8 Department of Neurosurgery, Xiangya Hospital, Central South University, Changsha, Hunan China

**Keywords:** Tregs, Heterogeneity, Fragility, Anti-tumor effects, Immunotherapy

## Abstract

Regulatory T cells (Tregs) expressing the transcription factor FoxP3 are essential for maintaining immunological balance and are a significant component of the immunosuppressive tumor microenvironment (TME). Single-cell RNA sequencing (ScRNA-seq) technology has shown that Tregs exhibit significant plasticity and functional diversity in various tumors within the TME. This results in Tregs playing a dual role in the TME, which is not always centered around supporting tumor progression as typically believed. Abundant data confirms the anti-tumor activities of Tregs and their correlation with enhanced patient prognosis in specific types of malignancies. In this review, we summarize the potential anti-tumor actions of Tregs, including suppressing tumor-promoting inflammatory responses and boosting anti-tumor immunity. In addition, this study outlines the spatial and temporal variations in Tregs function to emphasize that their predictive significance in malignancies may change. It is essential to comprehend the functional diversity and potential anti-tumor effects of Tregs to improve tumor therapy strategies.

## Background

Regulatory T cells (Tregs), a subgroup of CD4^+^ T cells that express the FoxP3 gene and have immunosuppressive properties, are found in high numbers in the tumor microenvironment (TME) of different types of malignancies [1, 2]. Tregs impede immune monitoring of tumors in healthy individuals and diminish the anti-tumor immune response in tumor patients, potentially resulting in tumorigenesis and progression [3]. In addition, Tregs support tumor cells survival by releasing growth factors and interacting with stromal cells [4]. Increased presence of Tregs is linked to decreased patient survival in several types of malignancies [5–7]. Tregs infiltration is associated with decreased recurrence-free survival (RFS) in non-small-cell lung cancer (NSCLC) patients [8]. Recently, with the widespread use of single-cell sequencing technologies, research has revealed the anti-tumor actions of Tregs in diverse TME. However, the mechanisms involved are not well understood.

Tregs’ immunosuppressive activity plays a dual role in both the development and advancement of tumors [9]. Originally, inflammation was believed to be the body’s immunological response against tumors. Tregs inhibit T effector cells (Teffs), natural killer cells, and dendritic cells by various mechanisms, which helps tumor cells avoid detection by the immune system [10–13]. Nevertheless, there is increasing evidence indicating that inflammation plays a significant role in the development and advancement of tumors [14]. The gut microbiota can cause long-lasting inflammation, which promotes the development of tumors [15]. Tregs suppress microbial-induced inflammation, therefore decreasing susceptibility to tumors [16]. Inflammatory cells and cytokines in the TME can enhance tumor angiogenesis, facilitating tumor growth and spread [17]. Tregs suppress the harmful inflammatory response and thus exhibit positive benefits in patients with several types of cancers, including colorectal cancer (CRC) and head and neck tumors (HNC) [16, 18–21].

However, not all Tregs cells are immunosuppressive, and recent research has found various subpopulations with anti-tumor effects. Researchers have categorized FoxP3^+^ Tregs into three subtypes according to the levels of FoxP3 and CD45RA expression. (1) Initial Tregs that have only weak inhibitory effects (FoxP3^low^CD45RA^+^); (2) effector Tregs with strong inhibitory and stabilizing functions (FoxP3^high^CD45RA^−^); (3) non-Tregs that mainly secrete inflammatory cytokines, thereby playing a role in promoting the body’s immune response non-Tregs (FoxP3^low^CD45RA^−^). The third group of FoxP3^low^ non-Tregs with high infiltration in CRC patients exhibited a superior prognosis [22]. New subpopulations of anti-tumor Tregs, such as Neuropilin-1^−^(Nrp1)^−^Tregs and TIGIT^−^Tregs, are continuously being discovered due to the use of single-cell sequencing technologies [23, 24]. Moreover, Tregs with high plasticity can transform into Th-like Tregs in certain situations, enabling them to produce specific pro-inflammatory cytokines. Tregs can develop T-bet expression and transform into Th1-like Tregs, which can then produce IFN-γ to help the immune system target tumor cells [25].

Tregs have the ability to offer anti-tumor effects in the TME, as shown by current research. There is disagreement over the exact anti-tumor mechanism, as different research have shown conflicting results. This review intends to comprehensively investigate the potential anti-tumor mechanisms of Tregs, such as suppressing pro-tumor inflammatory responses and boosting anti-tumor immunity. Additionally, this review will uncover the spatiotemporal heterogeneity of the prognostic value of Tregs in tumors and the underlyingmechanisms. Elucidating the anti-tumor mechanisms of Tregs and their functional heterogeneity will contribute to the refinement of anti-tumor immunotherapy.

## Differentiation and functional subgroups of Tregs

Tregs in the body are classified based on their origin as centrally developed thymic Tregs (tTregs) and peripherally induced Tregs (iTregs). tTregs are generated in the thymus and get activated by self-antigens provided by thymic epithelial cells. Mature tTregs are transported to the periphery where they mostly inhibit self-antigens. iTreg cells are primarily differentiated from naive CD4^+^ T cells in the thymus after receiving TCR signaling. They consist of various subgroups such as FoxP3^+^ iTregs, type 1 regulatory T cells (recognized by the phenotype CD4^+^ CD25^low^ CD45RB^low^), and Th3 (recognized by the phenotype CD4^+^ CD25^low^) [26]. Incomplete epigenetic foundation and functional instability characterize iTregs, which are closely associated with autoimmune disorders, cancer, and progression [27]. The TME contains chemicals such tumor antigens, IL-2, IL-10, TGF-β, and other soluble molecules that stimulate the T-cell receptor (TCR) and enhance the transformation of naive CD4^+^ T-cells into iTregs [28]. iTregs are the main kind of Tregs that enter tumors, despite the presence of both tTregs and iTregs. Tumor-infiltrating Tregs frequently exhibit a range of co-stimulatory molecules like CD27, ICOS, GITR, and OX40, along with co-inhibitory molecules such as CTLA-4, LAG-3, PD-1, and TIGIT [29].

**Fig. 1:**
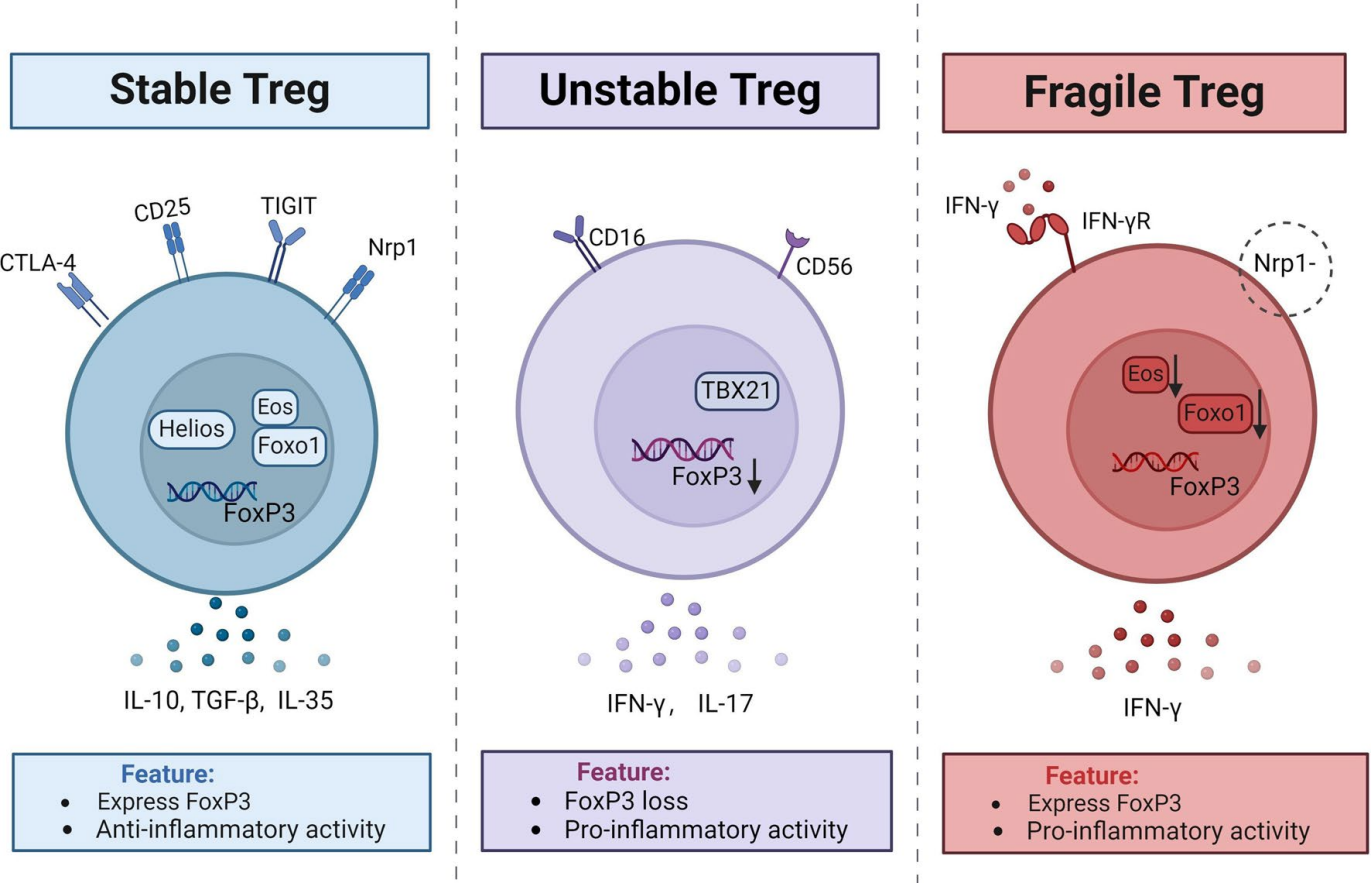
Three subgroups can be identified based on phenotype and function of Tregs. Stable Tregs: express FoxP3, have immunosuppressive function; Unstable Tregs: express low or no FoxP3 that secrete IFN-γ and can promote anti-tumor immunity; Fragile Tregs: express FoxP3 while secreting IFN-γ, and have anti-tumor activity [30]

Tregs display significant heterogeneity in phenotype and function. Researchers have classified Tregs into three categories according to their expression of FoxP3 and their inhibitory role. (1) Stable Tregs: express FoxP3, Nrp1, and Helios, exhibit immunosuppressive function, and are the predominant subgroup of Tregs; (2) Unstable Tregs: low or no expression of FoxP3 and secretion of IFN-γ, supporting anti-tumor immune responses; (3) Fragile Tregs: express FoxP3 and secrete IFN-γ, contributing to anti-tumor effects [30] (Fig. 1). Recently, scRNA-seq analysis has been commonly used to investigate the gene-level heterogeneity of Tregs and to identify crucial factors that influence their differentiation and function. This technique has revealed new subsets of Tregs with unique functional properties [31]. In head and neck squamous cell cancer (HNSCC), a specific subset of Tregs expressing tumor necrosis factor receptor (TNFR)^+^ and possessing inhibitory properties was discovered. The Tregs express TNFR genes and have large quantities of OX40, 4-1BB, and GITR molecules. Their existence is linked to a negative outlook for HNSCC patients. A transcriptional circuit, centered on the basic leucine zipper ATF-like transcription factor (BATF), was inferred by constructing a regulatory network for scRNA-seq.BATF is a crucial element that controls the transcription and activity of TNFR^+^ Tregs in the TME [32]. Various subpopulations of Tregs with varied phenotypes and functions are present in TME, but the amount of distinctions between these subtypes is not fully understood.

## Potential anti-tumor effects

As our understanding of Tregs grows, it has become evident that the belief that Tregs always lead to a poor tumor prognosis is too simplistic, especially in the later stages of the tumor. Tregs have been found to have multiple potential anti-tumor mechanisms [33]. Studies on various types of malignant tumors, such as CRC, HNSCC, and gastric cancer (GC), have shown a positive correlation between Tregs infiltration and prolonged survival of cancer patients [16, 18, 20, 34, 35]. The results indicate that Tregs have a positive impact on suppressing the development of tumors and the advancement of tumors. We summarized the potential mechanisms of anti-tumor effects based on Tregs functional subgroups and categorized them into two parts. (1) Specific subgroups of Tregs with altered phenotypes can increase the immune system's ability to kill tumors, primarily through Fragile Tregs, Unstable Tregs, and Th1-like Tregs; (2) Suppression of inflammatory reactions that support tumor growth, mainly through stable Tregs subpopulations.

### Tregs promote anti-tumor immune responses

#### Subpopulations of pro-inflammatory Tregs secrete cytokines with anti-tumor properties

**Fig. 2:**
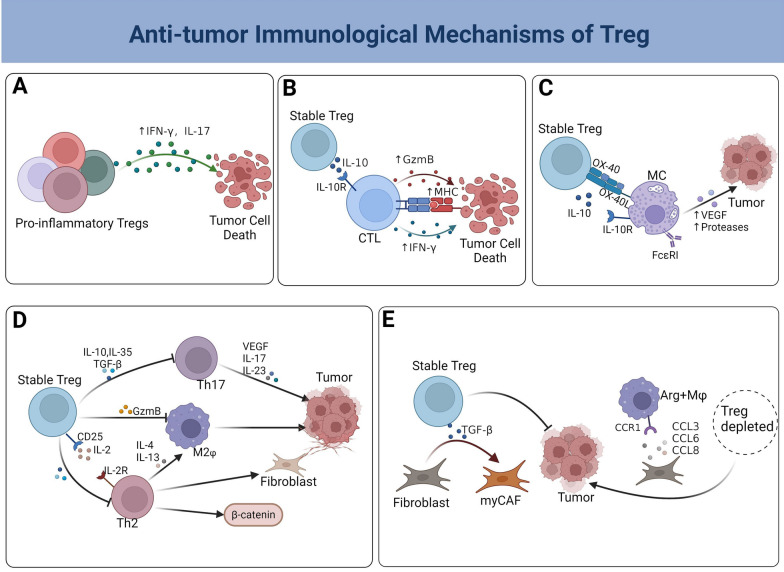
Potential anti-tumor mechanisms of Tregs. **A** Pro-inflammatory Tregs secrete inflammatory cytokines that have anti-tumor effects. **B** IL-10 secreted by Tregs promotes the activation of CD8^+^ T cells and induces the production of GzmB and IFN-γ, and further upregulates the expression of MHC molecules. **C** MCs release mediators that promote tumor metastasis and angiogenesis. Tregs decrease MC numbers and hinder their degranulation by releasing IL-10 and via the OX40-OX40L cell contact mechanism. **D** Th2 promotes aberrant expression of β-catenin, releases cytokines that activate M2 macrophages, and promotes tumor-associated fibrous capsule development, whereas Th17 releases cytokines that stimulate tumor angiogenesis and progression. Tregs suppress the pro-tumor inflammatory response induced by Th2, Th17, and M2 macrophages. **E** Tregs promote the differentiation of fibroblasts to myofibroblasts by releasing TGF-β, in turn suppressing tumor growth. Depletion of Tregs cells leads to an increase in the accumulation of fibroblasts and the recruitment of tumor-promoting Arg1^+^ Mφ cells

Tregs do not always function as suppressors, certain Tregs can change their function to become effector cells and produce inflammatory cytokines such as IFN-γ and IL-17, which can help destroy tumor cells. Single-cell sequencing technique has shown three distinct subpopulations of Tregs that exhibit varying secretory phenotypes (Fig. 2A).

##### Unstable Tregs

Tregs’ stability is characterized by the persistent presence of FoxP3, low methylation of the conserved non-coding sequence 2 (CNS2) gene, and the the maintenance of inhibitory function. Tregs with low or no FoxP3 expression are known as unstable Tregs or exTregs, and they have the capacity to secrete inflammatory cytokines such as IFN-γ and IL-17 [36]. FoxP3 expression maintenance is controlled by the conserved non-coding sequence 2 (CNS2), which binds both acute myeloid leukemia-1 (AML1)/Runx1 and FoxP3 in mature Tregs. FoxP3 interacts with AML1/Runx1 at CNS2, initiating DNA demethylation in the Tregs-specific demethylation region (TSDR), essential for Tregs stability [37]. In addition, IL-2 interacts to IL-2R on Tregs’ cell surface, maintaining FoxP3 expression via activating downstream STAT5 [38]. Under inflammatory conditions or in an IL-2-restricted environment, mature Tregs that divide may have their FoxP3 expression downregulated and be converted into unstable Tregs due to the deletion of CNS2 [39]. In addition to low expression of FoxP3, exTregs exhibit elevated expression of CD16, CD56, and CD127 compared to Tregs, along with increased levels of cytotoxicity and inflammatory genes including TBX21, NKG7, CCL3, CCL4, and CCL5 due to reduced FoxP3 expression [40]. To ascertain whether Tregs instability is inherent to a specific Tregs subpopulation or is a result of stochastic occurrences. Researchers utilized a genetically induced destiny mapping mouse model to validate the existence of an unstable subgroup of FoxP3^+^ Tregs in vivo, leading to the generation of ex-FoxP3 cells. Eliminating the unstable fraction in vivo increased the stability of Tregs [23]. A study revealed that Tregs in CRC patients exhibit functional heterogeneity, with both FoxP3^high^ Tregs and FoxP3^low^ Tregs subpopulations identified. The researchers CRC into two categories, A and B, depending on the FoxP3^low^ Tregs percentage. Type B displayed a substantial upsurge in the FoxP3^low^ Tregs percentage. FoxP3^low^ Tregs in type B CRC lose their immune suppression capabilities, secrete IL-17 and IFN-γ, and confer a favorable prognosis for patients [22]. Differences in gut bacteria and their production of cytokines IL-12 and TGF-β contribute to varying ratios of FoxP3^low^Tregs and FoxP3^high^ Tregs in different types of colon cancer. IL-12 and TGF-β combined enhance the proportion of FoxP3^low^Tregs, resulting in the activation of genes involved in immunological and inflammatory reactions [41]. The above suggests that accurate assessment of Tregs and exTregs cells is necessary in tumor immunity.

##### Fragile Tregs

Recent studies reported the existence of a fragile Tregs phenotype in the TME that produces IFN-γ and exhibits anti-tumor effects [30, 42–44]. Several elements, including Nrp1, Foxo1, and Eos, are essential in maintaining the stability of Tregs [45]. However, the type I IFN/IFNAR1 pathway can induce the transformation of Tregs to fragile Tregs [42]. The majority tumor-infiltrating Tregs express Nrp1, which enhances their immunosuppressive role by interacting with Semaphorin 4A (Sema4A) [46]. Nrp1^−^ Tregs not only lose their immunosuppressive role, but also become active participants in the anti-tumor immune response. In both mouse models and humans, deficiency of Nrp1 results in fragility of Tregs and an increase in IFN-γ secretion. This increased secretion affects neighboring Tregs, destabilizing their suppressive phenotype. In hypoxic conditions, the impact is further intensified by the increase in HIF1-α. IFN-γ is mainly produced by Nrp1^−^ Tregs in the TME. This release preserves the anti-tumor function of fragile Tregs and enhances the efficacy of PD-1 inhibitors on malignancies [47]. Although IFN-γ produced by other immune cells can also contribute to the fragility of Tregs, it is not the primary source. The fragile Tregs subset is associated with a better prognosis in patients with metastatic melanoma and HNSCC. Nrp1 is not required to inhibit autoimmunity and maintain immunological homeostasis [47, 48]. Thus, directing Tregs towards Nrp1^−^ Tregs shows promise as a treatment strategy that improves prognosis without triggering an overwhelming autoimmune reaction [49]. Additionally, the reduction of Foxo1 and Eos in Tregs can create a fragile Tregs profile, producing substantial levels of IFN-γ, which can improve clinical outcomes [50]. The presence and potential utility of fragile Tregs in malignancies for preventive or therapeutic purposes are still a subject of controversy and uncertainty.

##### Th-like Tregs

Tumor-infiltrating Tregs exhibit plasticity and have the ability to modify their features to function similarly to effector T cells while maintaining high amounts of FoxP3, known as Th-like Tregs [51]. Transcription factors' expression may influence the phenotypic and functional specialization of Tregs, in which multiple Th-like Tregs subpopulations have been reported [52]. When exposed to IL-12, FoxP3^+^ Tregs express the transcription factor T-bet, causing them to transform into Th1-like Tregs (T-bet^+^IFNγ^+^FoxP3^+^ Tregs) [53]. They lose their inhibitory function and secrete the pro-inflammatory cytokine IFN-γ [54]. Th1-like Tregs utilize an active PI3K/AKT/FOXO pathway as their main mechanism for IFN-γ production [55]. In vitro studies show that T-bet^+^ Tregs are capable of producing IFN-γ independently of IL-12, suggesting that T-bet expression alone is sufficient to trigger IFN-γ production. IL-12 could enhance this impact [56]. An increased number of activated T-bet^+^ Tregs were seen in cases of oropharyngeal squamous cell carcinoma (OPSCC) generated by human papillomavirus (HPV), and this was associated with longer survival rates [57]. In addition, Th17-like Tregs (IL-17^+^RORγt^+^FoxP3^+^ Tregs) were identified in TME. These Tregs express RORγT and produce IL-17 when stimulated by IL-6 and TGF-β [58]. The current evidence on the involvement of IL-17 in cancer is inconclusive. One viewpoint suggests that the production of IL-17 caused by microbes promotes tumor growth, as explained before. Another theory proposes that IL-17 boosts the body's defense against tumors by orchestrating the attraction and stimulation of natural killer (NK) cells and T cells through increasing the production of chemokines (CCL2 and CXCL10) [59]. In this context, Th17-like Tregs secreting IL-17 within tumors may be advantageous for patients with malignancies [60].

#### FoxP3^+^ Tregs are associated with a more prominent role of CTLs in TME

Controversy exists about the traditional view that FoxP3^+^ Tregs consistently exhibit immunosuppressive functions. FoxP3^+^ Tregs presence may indicate increased infiltration and toxic effects of cytotoxic T lymphocytes (CTLs). Several studies have demonstrated that tumors with a high quantity of FoxP3^+^ Tregs also have a large presence of other immune cells such as CD8^+^ T cells and proliferative immune cells [61, 62]. For example, there is a significant correlation between FoxP3^+^ tumor-infiltrating lymphocytes (TILs) and total CD8^+^ T cells infiltration in triple-negative breast cancers [63]. However, the causal relationship between FoxP3^+^ Tregs and CD8^+^ T cells infiltration in TME remains controversial to date. Research indicates that IFN-γ from CD8^+^ T cells in tumors can stimulate dendritic cells (DCs) to release more CXCL9, which then draws a significant number of CXCR3^+^FoxP3^+^ Tregs that oppose this effect [64]. Conversely, there are studies that suggest FoxP3^+^ Tregs promote the infiltration of T cells into the peripheral circulation within the TME [63]. T cell extravasation from blood vessels requires interaction with endothelial selectins (CD44, CD62L, or PSGL) for initiating rolling within the vasculature. FoxP3^+^ Tregs are capable of expressing these endothelial selectins [65]. More importantly, ligands for most of the chemokine receptors that mediate CD8^+^ T cell extravasation, such as CCR5, CXCR3, and CXCR6, are also expressed by FoxP3^+^ Tregs [66–68]. Additionally, in ER-negative breast cancers, elevated levels of FoxP3^+^ Tregs exhibited a strong correlation with gene expression profiles characteristic of differentiated CTLs [63]. Thus, the positive correlation between FoxP3^+^ Tregs and CD8^+^ T cells in tumors may also occur due to the increase of CD8^+^ T cell infiltration and differentiation induced by FoxP3^+^ Tregs.

Additionally, studies have shown that patients with gastrointestinal malignancies including both FoxP3^+^ Tregs and CD8^+^ T cells have better survival rates than individuals with gastrointestinal tumors that do not have these cell types [69–71]. Multifactorial Cox regression analysis demonstrated that FoxP3^+^ Tregs served as an independent and favorable prognostic factor for patients with CD8^+^ T cell infiltration in HER2^+^/ER^−^ breast cancer. Patients with higher CD8^+^ T cell levels who also had increased FoxP3^+^ Tregs infiltration showed a 52% greater chance of survival than those with low levels of FoxP3^+^ Tregs infiltration [72]. It can be inferred that Tregs may work together with CD8^+^ T cells to enhance anti-tumor immunity. Relevant studies have shown that IL-10 produced by Tregs interacts with IL-10R on CD8^+^ T cells to enhance their activation and infiltration. This interaction also triggers the production of granzyme and IFN-γ by triggering the phosphorylation of STAT1 and STAT3. In addition, IL-10 can stimulate the production of IFN-γ, which in turn increases the expression of major histocompatibility complex (MHC) molecules, enhancing the presentation of tumor antigens [73–75] (Fig. 2B). The findings indicate that Pegylated IL-10 has therapeutic potential for solid malignancies [74]. Taken together, the observation that high levels of tumor-infiltrating Tregs, in contrast to their known immunosuppressive function, can actually boost the host immune response, leading to a more potent effect of CTLs. This phenomenon helps explain why tumor patients may have a better prognosis due to Tregs.

### Regulatory role of stable Tregs in tumor-promoting inflammation

In vivo, immune responses against tumors occur alongside inflammation that supports the development and advancement of tumor [76]. Chronic inflammation from different sources can initiate tumor formation, such as hepatitis B and C infections leading to liver cancer, HPV infection causing cervical cancer, and inflammatory bowel disease contributing to CRC [77–79]. Moreover, inflammatory cells and their mediators heighten the probability of genetic instability [80]. Additionally, they induce tumor angiogenesis and chemoresistance, promoting the proliferation and invasion of tumor cells [81]. FoxP3^+^ stable Tregs, a type of immunosuppressive cells, demonstrate anti-tumor benefits by inhibiting tumor-promoting inflammation.

#### Inhibition of pro-tumor escape of mast cells (MCs)

MCs support tumor growth by creating an inflammatory TME to facilitate immune escape. Stable Tregs can partially improve tumor prognosis by inhibiting this process [82]. MCs originate from hematopoietic stem cells in the bone marrow and possess several tiny secretory granules. MCs release inflammatory mediators such as chymotrypsin and trypsin during degranulation, which can break down extracellular matrix (ECM) components and facilitate tumor cell implantation [83]. Additionally, Moreover, MCs secrete substances including VEGF-A, VEGF-B, fibroblast growth factor, histamine, heparin, and stem cell factors that promote tumor angiogenesis [84, 85]. Stable Tregs can impede the differentiation of MCs and obstruct their degranulation via production of soluble factors and contact-dependent mechanisms. Introducing stable Tregs into mice with existing polyps resulted in reduced polyp size and decreased infiltration of MCs, a course of action that necessitates the involvement of IL-10 [86]. IL-10 inhibits degranulation of MCs by suppressing expression and signaling of IgE receptor (FcεRI) in MCs. Moreover, stable Tregs inhibit degranulation of MCs by interacting with them via OX40-OX40L, which directly inhibits Ca^2+^ influx following FcεRI activation in MCs [87]. Stem cell factor (SCF) plays a crucial role in the expansion and maturation of MCs, while IL-17 enhances SCF production [88]. Stable Tregs can inhibit MCs differentiation by downregulating IL-17. Inhibition of MCs differentiation and degranulation by Tregs is beneficial for tumor patients (Fig. 2C).

#### Inhibition of tumor-promoting inflammatory responses induced by Th2/Th17 cells

CD4^+^ T cells play a crucial role in the immune system by coordinating adaptive immunological responses [89]. The role of Th2/Th17 cells in malignancies is still a topic of debate, as they are a subgroup of CD4^+^ T cells [90, 91]. Several studies indicate that they play a role in stimulating tumor growth. Stable Tregs can limit pro-tumorigenic inflammatory responses caused by the activation of Th2/Th17 cells. This inhibition occurs through the release of immunosuppressive substances like IL-10 and TGF-β, as well as through other methods [91]. Diverse transcriptional patterns of Tregs inhibit specific CD4^+^ Th cells. Th17-like Tregs expressing STAT3 inhibit Th17 cell response, while Th2-like Tregs expressing IRF4 suppress Th2 cell response [92, 93].

Th2 cells have a poor correlation with prognosis in certain types of malignancies. For instance, pancreatic cancer patients with high levels of Th2 cells infiltration experience reduced survival rates [94]. First, Th2 cells promotes polarization and function of M2-tumor-associated macrophages (TAMs), resulting in an inflammatory TME that leads to a worse prognosis [95]. Th2 cells alternatively activates M2-TAMs through secretion of IL-4 and IL-13 cytokines [96]. Th2 cells stimulate M2-TAMs cathepsin protease activity in tumors by releasing IL-4, leading to enhanced tumor invasiveness and proliferation in the IL4-/-RT2 animal model and in vitro [97]. Secondly, Th2-related cytokines are prevalent in colitis and encourage abnormal β-catenin expression, leading to tumor formation [98]. Nuclear β-catenin accumulation was observed in half of CRC patients, and Wnt/β-catenin signaling dominated the early stages of sporadic colorectal cancer (SCC) [99]. The fibrotic capsule around the tumor can impede the effectiveness of drugs and immune cells in killing the tumor. Th2 cells contribute to the formation of this capsule by aiding fibroblasts in promoting fibrosis and sustaining the long-term effects of pro-fibrotic type 2 inflammation [100, 101]. This ultimately shields the tumors or metastases from harm. Reduced Treg levels result in the stimulation and proliferation of Th2 cells. Stable Tregs promote the death of Th2 cells by CTLA-4 expression and competing for IL-2, which plays a crucial role in inhibiting Th2 cells from promoting tumor growth and metastasis [102].

Stable Tregs counteract the pro-tumorigenic, cancer-progressing, treatment-resistant, and metastatic impacts of Th17 cells and their released cytokines. IL-17 produced by Th17 cells triggers the activation of anti-apoptotic proteins including Akt, Erk, mTOR, Bcl-2, and Bax, along with activating factors such as NF-κB, STAT, and AP-1. These factors promote the growth and regeneration of cancer stem cells and are linked to the onset of different cancer forms [60]. Enterotoxigenic Bacteroides fragilis (ETBF), a common microorganism in the human intestinal tract, directly induces the activation of STAT3 in the colon, leading to the promotion of Th17 cell differentiation [103]. Similarly, Th17 polarization is observed in gastric cancer induced by *H. pylori* [104]. Inhibiting IL-17 and IL-23, which are cytokines that boost the Th17 immune response, prevents tumor formation caused by ETBF [105]. Stable Tregs rely significantly on IL-10 to prevent microbial promotion of carcinogenesis by activating STAT3 and suppressing Th17 [106, 107]. On one hand, Tregs expressing STAT3 increase the expression of surface inhibitory chemicals, cytokines, and chemokine receptors to inhibit Th17 cells and promote the spatial proximity of Tregs and Th17 cells. On the other hand, STAT3 expression in Tregs curtails the expression of soluble mediators of Th17 differentiation [92]. Within developed tumors, IL-17 triggers the production of matrix metalloproteinases (MMPs). MMPs release and activate VEGF ligands, which are crucial for tumor metastasis [108]. IL-17 influences the suppressive immune cell milieu and stimulates cancer cell growth by triggering the production of chemokines (CXCL8, CXCL5, and CXCL6) and cytokines (G-CSF) [109]. Stable Tregs suppress the proliferation and generation of IL-17, IL-22, and CXCL8 by Th17 cells, therefore reducing the inflammatory response that supports tumor growth [110]. In addition, stable Tregs can inhibit the expression of matrix metalloproteinases through TGF-β signaling, resulting in decreased tumor metastasis [111, 112]. Stimulation of ST2/IL-33 pathway in stable Tregs enhances their quantity and function, while decreasing IL-17 production [113]. In tumors, the immune system can react to both tumor and microbial antigens, and stable Tregs can preferentially attenuate this tumor-associated inflammatory antimicrobial response [114]. The high infiltration of Tregs appears to positively correlate with improved prognosis in tumors with a high microbial pathogen content. Depletion of Tregs in pancreatic cancer resulted in an increase in CD4^+^ T cells and accelerated oncogenesis [115, 116]. This evidence is reinforced by the outcome that in an Apc^Min/+^ mouse model of intestinal cancer, Tregs immunotherapy effectively hindered the formation of intestinal adenomas and prompted the reduction of pre-existing adenomas [117]. Therefore, we hypothesize that stable Tregs can impede the advancement and growth of specific tumors by quelling the Th2/Th17 inflammatory response (Fig. 2D).

#### Inhibition of tumor-associated macrophages (TAMs) mediated promotion of tumors

Stable Tregs, as a type of immunosuppressive cells, can also hinder TAMs that have pro-tumorigenic effects, leading to enhanced tumor suppression. TAMs, which are a significant element of TME, frequently shows a positive correlation with the depth of tumor infiltration and clinical staging [118]. Research indicates that the infiltration of M1-TAMs are linked with improved survival rates among those with GC, while M2-TAMs count and total TAMs present as risky prognostic factors [119]. M2-TAMs play a significant role in tumor angiogenesis, promoting tumor cell growth, infiltration, metastasis, lymphatic vessel and lymph node metastasis, and mediating immunosuppression [120]. Tregs can inhibit the origin of TAMs [121, 122]. TAMs are primarily recruited from peripheral blood mononuclear cells through chemokines [123]. TAMs are predominantly recruited from peripheral blood mononuclear cells through chemokine signaling. In a human cell model induced by lipopolysaccharide (LPS), stable Tregs restrain monocyte survival by secreting soluble factors that participate in the Fas-mediated apoptosis pathway [121]. Certain subpopulations of Tregs have been found to inhibit TAMs directly. Tr1 cells are a subtype of Tregs that do not express CD25 or FoxP3 and are defined as CD4^+^FoxP3^−^ Tregs. Tr1 secretes high levels of IL-10 and TGF-β to regulate T cell responses [124]. Significant secretion of granzyme B and perforin was reported when Tr1 cells were co-cultured with TAMs in an in vitro experiment. Blocking Tr1 cell granzyme B and perforin led to a notable decrease in TAM-specific cell death, allowing the tumor cell lines to thrive in this setting. The data indicate that Tr1 cells eradicate TAMs using a granzyme B and perforin-dependent mechanism, which enhances tumor growth [125].

#### Promoting differentiation of carcinoma-associated fibroblasts (CAFs) to myCAFs

CAFs can be activated by resident tissue fibroblasts or transdifferentiated from non-fibroblast cells, such as adipocytes and epithelial cells, due to stimulation from TME [126]. CAFs play a crucial role in promoting the progression of tumors and metastasis through the production of soluble factors, including cytokines, chemokines, and the extracellular matrix. Such factors promote the growth of cancer cells, suppress immune responses that aim at tumors, remodel the ECM, influence resistance of tumor cells to drugs, and promote angiogenesis [127]. Inflammatory cancer-associated fibroblasts (iCAFs) and myofibroblastic cancer-associated fibroblasts (myCAFs) are two prevalent CAF types with distinct and at times opposed roles [128]. IL-1 initiates JAK/STAT activation leading to the production of iCAFs. Conversely, TGF-β counters this process and facilitates the conversion of myCAFs [129]. The overall function of myCAFs expressing α-smooth muscle actin (SMA) is to suppress tumor growth, and there is a significant correlation with favorable patient survival [128, 130]. In recent years, research has identified that Tregs cells act as regulators of myCAFs. Furthermore, TGF-β dependent action of stable Tregs has been found to promote the differentiation of CAFs into tumor-suppressor α-SMA^+^ myCAFs [131]. Pancreatic ductal adenocarcinoma (PDAC) is linked with significant fibrosis and stromal myofibroblasts. Depleting Tregs in a mouse model of pancreatic adenocarcinoma leads to CAFs reprogramming, evident by a decrease in α-SMA expression and loss of tumor-suppressing myCAFs, resulting in a massive accumulation of tumor-promoting CAFs. CAFs release chemokines (CCL3, CCL6, CCL8) that stimulate the recruitment of immunosuppressive arginase1^+^ positive macrophage (Arg1^+^Mφ) through its interaction with CCR1 [132]. The accumulation of activated CAFs within tumors results in the production of ECM-degrading proteases, which release growth factors and cytokines, ultimately promoting the motility and invasion of cancer cells [133]. In addition, the depletion of Tregs results in the upregulation of the expression of immunosuppressive genes, including arginase1 and CD274 (PD-L1) [132]. The depletion of Tregs leading to a decrease in TGF-β may partially explain the increase in carcinogenesis, suggesting a direct effect. Additionally, this study analyzed tissue levels and found that the positive effect of Tregs depletion, which results in an increase in CD8^+^ T cells, was negated by an increase in other CD4^+^ Th cells, immunosuppressive myeloid cells, and fibroblast populations, ultimately failing to inhibit tumor growth [116, 131] (Fig. 2E).

## Spatiotemporal heterogeneity of the prognostic value of Tregs in the TME

The prognostic significance of Tregs in tumors remains contentious, with diametrically opposed outcomes even within the same tumor type. Certain studies suggest a correlation between heightened Tregs levels and shorter overall survival (OS) and disease-free survival (DFS) in patients with pancreatic cancer [134]. Another study demonstrated that a significant infiltration of Tregs extends survival time in individuals diagnosed with pancreatic cancer [115]. The prognostic controversy of Tregs may relate to their functional heterogeneity at different stages and in different regions of the tumor. The plasticity of Tregs indicates their complex and variable function, demonstrated by the loss of FoxP3 expression and the acquisition of Th-like functions [135, 136]. The composition and functional status of TME vary significantly across tumor types, locations, and stages. Tregs’ phenotype and function are affected by various stimuli, leading to the expression of both pro-tumor and anti-tumor activity [137]. Further investigation is required to determine how Tregs regulate anti-tumor immune responses under varying conditions due to the functional heterogeneity of Tregs and the complexity of TME.

### Tregs display functional heterogeneity across various types of tumors

**Fig. 3:**
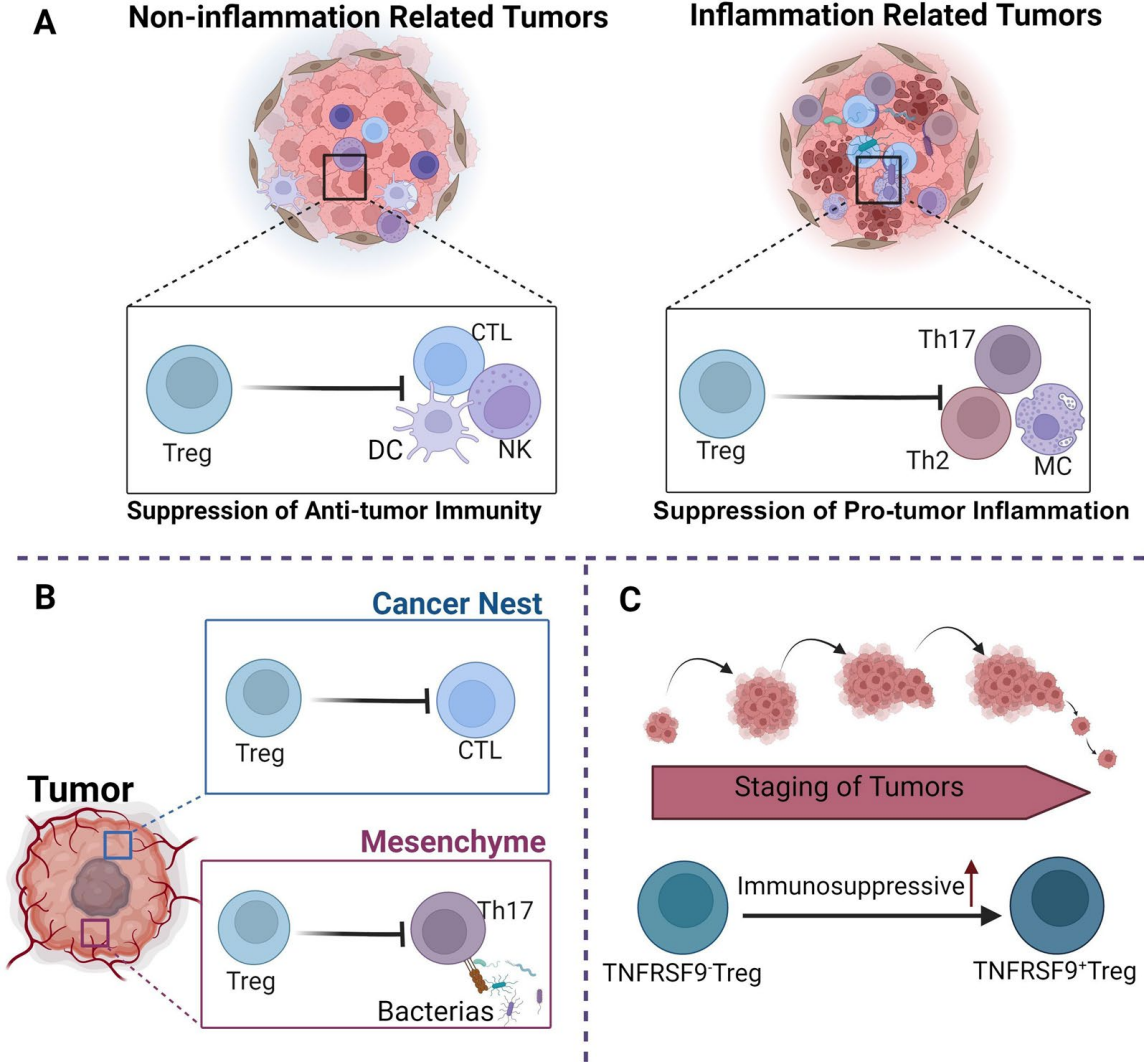
Spatiotemporal heterogeneity of Tregs function in TME. **A** In non-inflammatory tumors, Tregs mainly inhibit anti-tumor immunity. Conversely, in inflammatory tumors, Tregs tend to improve prognosis by suppressing tumor-promoting inflammation. **B** There exist variances in the function of Tregs in the tumor nests as compared to those in the mesenchyme. **C** As the tumor progressed, TNFRSF9^−^ Tregs transformed into TNFRSF9^+^ Tregs, leading to a gradual enhancement of their immunosuppressive abilities

The published literature shows that Tregs are frequently present in tumors, such as lung cancer and liver cancer, with unfavorable clinical outcomes. On the other hand, the presence of Tregs in chronic inflammation-related tumors, such as CRC and HNC, typically indicates a better prognosis. This suggests that there may be variation in the function of Tregs in different tumor tissues. Tumors can be classified into “immune desert”, “immune exclusion”, and “inflammatory” categories based on the number of infiltrated CD8^+^ T lymphocytes [138, 139]. Numerous studies have demonstrated that “inflammatory” tumors with high stable Tregs infiltration show better survival rates. For instance, a substantial immunohistochemical study found that high-density Tregs had a better prognosis in the HER2^+^/ER^−^ category of breast cancers, but only if accompanied by a high infiltration of CD8^+^ T cells [72]. In the "inflammatory" group of HNC, low stromal Tregs counts were correlated with larger tumor size (p < 0.001) and more lymph node metastases (p = 0.039). Conversely, higher Tregs counts were associated with more undifferentiated tumors (p < 0.001) and more lymph node metastases (p = 0.039) in the “immune desert” group. Additionally, Tregs counts in the “immune desert” group were linked to more undifferentiated cancers, including tumor grade (p = 0.018) [140]. The improved prognosis observed for Tregs in “inflammatory” tumors may be due to their suppression of the predominantly pro-inflammatory tumor response. However, in non-inflammation-associated tumors, Tregs hindered the anti-tumor effects of CTLs, dendritic cells, and NK cells (Fig. 3A). Further, functional differences of Tregs in different tumors have been directly observed by researchers. For instance, the functions of FoxP3^+^ Tregs varied between gastric signet-ring cell carcinoma (GSRCC) and non-GSRCC. In GSRCC, Tregs demonstrated increased naive characteristics (represented by CCR7 and TCF7), limited inhibitory functions (represented by PRDM1, IKZF2, and EGR1), and migratory capacity (represented by Rgs1) compared to non-GSRCC, resulting in different prognostic values [141]. Thus, certain inflammatory conditions and types of tumors can greatly impact both the phenotype and functionality of Tregs in TME.

### Functional diversity of tregs in various regions of the tumor

Tumor-infiltrating Tregs located in the center and margins may lead to varied patients prognosis. In CRC, Tregs situated in the tumor mesenchyme result in a better prognosis, whereas Tregs residing in the tumor nests and infiltrating margins are linked to unfavorable clinical outcomes [142, 143]. There is evidence to suggest a correlation between the varying functions of Tregs in different TME and the resultant inconsistency observed. Tregs in the mesenchyme exhibit an inhibitory effect mostly on Th17 cells mediated inflammatory anti-microbial responses, leading to indirect suppression of tumor growth. In contrast, Tregs infiltrating cancer nests and tumor margins have the opposite impact, primarily by directly suppressing the anti-tumor immune response of effector T cells [144] (Fig. 3B). It has been observed that Tregs in the epithelial area of CRC have improved clinical outcomes when located near CD8^+^ T cells, whereas the opposite holds true for Tregs in the stromal region [145]. This phenomenon is observed in patients with NSCLC, where a substantial presence of Tregs within the core tumor area predicts reduced overall survival independently. This adverse effect may be mitigated by enhancing the local CD8^+^ T cells [146]. The role of Tregs within distinct regions of the tumor may vary and result in varying prognoses. Nevertheless, the exact mechanisms necessitate further clarification.

### Differences in the function of Tregs at different tumor stages

The role of FoxP3^+^ Tregs in patients with tumors and their impact on prognosis is a contentious topic that may be affected by the tumor’s clinical stage [147]. In a retrospective study of gastric cancer, tumors were categorized by TNM staging. Researchers discovered that patients with high FoxP3^+^ Tregs infiltration during the early stages (I–II) had a greater chance of surviving for 5 years, while those in stages III–IV had a worse prognosis [148]. This study showed that FoxP3^+^ Tregs is an independent and favorable prognostic factor in early stages of gastric cancer. The findings highlight the complexity of FoxP3^+^ Tregs function in various tumor stages, which may relate to the fact that Tregs mainly suppress pro-tumor inflammation in early stages of tumors, but hinder tumor killing by effector T cells in advanced stages. Tumor progression can impact Tregs function, leading to the creation of a pro-tumorigenic microenvironment. Single-cell sequencing has revealed a standard developmental trajectory of Tregs in cancer, moving from a resting state (TNFRSF9^−^ Tregs) to an activated state with increased suppressive capacity (TNFRSF9^+^ Tregs) [149] (Fig. 3C). Thus, the prognostic variances of Tregs in different tumor stages are linked to the modified functional status of Tregs. However, few studies have examined the correlation between tumor stage and Tregs function, making it necessary to further investigate the relationship between Tregs and prognosis in different tumor stages, as well as its underlying mechanisms in the future.

## New advances and therapies for targeting Tregs against tumors

With extensive analysis of the role of Tregs in tumors and the advancement of precision therapy, significant progress has been achieved in anti-tumor therapy strategies that target Tregs. First, targeting tumor-infiltrating Tregs (TI-Tregs) while preserving peripheral Tregs (P-Tregs) effectively prevents autoimmune reactions. Single-cell RNA sequencing analyses revealed that the simultaneous loss of colonic tissue Tregs cells during antitumor application of PI3Kδ inhibitors causes colitis by impairing the ability to safeguard tissue homeostasis. This points to a specific mode of action for the emergence of immune-related adverse events (IRAEs) [150]. Precisely targeting TI-Tregs proves effective in avoiding adverse effects. TI-Tregs express a broad range of specific genes, including CCR8, which is a candidate therapeutic target playing a vital role in the recruitment of Tregs to sites of tumor and inflammation [151]. By using a monoclonal antibody against CCR8, tumor Tregs were selectively eliminated, thereby curing established tumors in mice. There were no instances of tissue inflammation, splenomegaly, or circulating autoantibodies observed with the use of the CCR8 monoclonal antibody, unlike with the indiscriminate depletion of all Tregs [152]. Several drugs aimed at targeting CCR8 are presently in development, including BMS-986340, which is a CCR8-targeting antibody drug in clinical trials, with a Phase 1/2 clinical trial presently underway [153]. Furthermore, an examination compared TI-Tregs and P-Tregs in 36 patients from 4 malignancies and identified 17 master regulators (MRs) that are determinants of the transcriptional status of TI-Tregs. To identify drugs that specifically hinder the infiltration of Tregs in the TME, the researchers initially analyzed the impact of a range of 1554 FDA-approved drugs on the viability of human-origin P-Tregs. Gemcitabine had a dose-dependent effect on reducing Tregs viability. Low doses of gemcitabine considerably decreased Tregs in tumors, restraining the transcriptional activity of TI-Tregs MR (including TRPS1) in various tumor types, while not affecting the number of splenic Tregs. This confirmed that the inhibitory effect of gemcitabine was restricted to TI-Tregs [154]. Secondly, it is crucial to preserve the function of anti-tumor cytotoxic Tregs, as Tregs are a heterogeneous group. Therefore, caution should be exercised to ensure the preservation of Tregs' anti-tumor function. As previously mentioned, various subpopulations of Tregs with different functions exist in tumors, some of which are phenotypically comparable to conventional T cells that not only lack an inhibitory effect on the immune system but can also enhance the killing effect on tumors [155, 156]. Promoting tumor killing is more effective than suppressing the immune system. Therefore, targeting specific subpopulations of Tregs that promote tumors, rather than all Tregs, can improve prognosis. However, there is a shortage of large-sample single-cell studies on Tregs that can define detailed subpopulations of Tregs and their corresponding markers and functions. Finally, targeting Tregs for anti-tumor purposes must be done with caution due to their spatiotemporal heterogeneity in the TME, taking into account both tumor type and stage.

Existing anti-tumor therapies that target Tregs are often achieved by depleting or inhibiting Tregs’ function and have limitations. Promoting Tregs towards an anti-tumor phenotype that exploits their auto-reactivity against tumors increases the inflammatory response within tumor tissue, resulting in enhanced anti-tumor immune response. This approach is an ideal treatment modality [155]. The subpopulation of Tregs that secrete IFN-γ and other cytokines, also known as the Tregs anti-tumor phenotype, is predominantly represented by unstable Tregs, fragile Tregs, and Th1-like Tregs. Therapeutic approaches that widen the local unstable catchment area of the tumor, such as the use of the small molecule nonpeptide p300 inhibitor, C646 (p300i), have been demonstrated to boost the body’s anti-tumor immune response [157]. Induction of Tregs fragility not only enhances antitumor activity in the TME, but also maintains peripheral tolerance and reduces iRAEs. The Nrp1-SEMA4A axis stabilizes intra-tumor Tregs and helps maintain their inhibitory function. The Nrp1 antagonist, Fc(AAG)-TPP11, selectively inhibits the function and stability of Nrp1^+^ Tregs in tumors, thereby facilitating tumor clearance [49]. Moreover, Tregs fragility can be induced by the IFN-I/IFNAR1 pathway, and the expression of IFNγR1 on Tregs is necessary for this effect. Inhibitors of p38α and sumoylation can promote Tregs fragility and constrain tumor growth by sustaining IFNAR1 levels [42]. Previously, it was believed that IL-12 was the primary factor driving Tregs to Th1-like Tregs, however, new discoveries on the PI3K/AKT axis as the primary signaling pathway regulating Th1-like Treg production have made Th1-like Treg production viable in many disease settings. These findings also imply the presence of other environmental factors that induce Th1-like Treg production [55]. Exploration of molecules that facilitate Th1-like Tregs polarization can potentially aid in directing tumor therapy. While the clinical significance and potential of converting Tregs to anti-tumor phenotypes is still being investigated, given their relevance to clinical outcomes, this therapeutic approach is promising.

Although immune checkpoint inhibitors (ICIs) have shown significant effectiveness against various malignant tumors, a significant proportion of patients are still unresponsive, resistant, or even experience hyperprogression when undergoing ICIs therapy, and the underlying mechanism behind this phenomenon remains unknown [158]. PD-1/PD-L1 inhibitors are a frequently used class of ICIs. The efficacy of PD-1/PD-L1 inhibitors is often reduced by the presence of Tregs. This effect is further enhanced by alterations in the number and function of Tregs following application of PD-1/PD-L1 inhibitors [159]. Tregs express PD-1, which plays a vital role in their immunosuppressive and developmental functions [160]. Anti-PD-1/PD-L1 antibodies lead to enhanced TCR signaling and CD28 signaling in Tregs, activating Tregs and increasing the proportion of Tregs within the tumor. In a mouse model of tumor-induced resistance to anti-PD-L1 therapy, the investigators observed that CD8^+^ T activation was much less and lagged behind that of Tregs. Rather than promoting Tregs proliferation overall, mainly fostered the proliferation of a subpopulation of PD-1^+^ Tregs, with a minor effect on PD-1^−^ Tregs. Moreover, Tregs treated with anti-PD-L1 increased the expression of key signature genes associated with inhibitory functions, including IL-10, TIGIT, and ICOS. This could potentially be a crucial factor in the failure of treatment and hyperprogression using PD-1/PD-L1 inhibitors. Studies indicate that Tregs elimination can efficiently reverse anti-PD-L1 treatment resistance [161]. In an unpublished phase II clinical trial of initial treatment for NSCLC with high PD-1 expression, researchers reported a significant clinical benefit in PFS and OS in patients treated with a combination of the TIGIT inhibitor, tirilizumab, compared to PD-L1 monoclonal antibody atirizumab monotherapy. However, Tregs were discovered in an ICIs responsive mouse tumor model to acquire a Th1-like transcriptional signature upon tumor entry, i.e., upregulation of T-bet and certain pro-inflammatory disease-related genes. Homology analysis revealed the existence of a similar subpopulation in human lung cancer, which is also enriched in tumors that respond to ICIs [162]. The study found that disruption of the CARD9-BCL10-MALT1 (CBM) signaling complex in TI-Tregs from mice resulted in loss of their inhibitory function and a shift to a pro-inflammatory, IFN-γ-secreting phenotype, leading to enhanced efficacy of anti-PD-1/PD-L1 antibodies [163]. Therefore, the combination of anti-PD-1/PD-L1 antibody and targeted Tregs therapy may be more efficacious in hindering tumor growth.

## Future perspectives of Tregs in TME

Many studies frequently use solely FoxP3 or just one or two additional markers (e.g., CD25^high^ and CD127^low^) as substitutes to identify Tregs, but this approach lacks accuracy and precision [164]. FoxP3 is also transiently expressed on effector T cells and tumor cells in humans [165]. In addition, Tregs comprise distinct subgroups with different phenotypes and functions, FoxP3 or other markers used so far are not sufficient to distinguish between functional subgroups [166]. Therefore, Previous studies may have been confounded by non-Tregs and other functional subpopulations of Tregs. scRNA-seq has been extensively utilized in immunological research, substantially enhancing our comprehension of different Tregs subpopulations by resolving heterogeneity of immune cell subpopulations at the single-cell level [167]. In the future, it is recommended to search for more specific and precise markers for subgroups of Tregs and to analyze potential connections and developmental differentiation relationships, between various subgroups as a whole. In addition, the use of spatial genomics will provide a more thorough comprehension of the distribution of various Tregs subgroups within the tumor. Meanwhile, integrating single-cell sequencing data from tumor patients at varying stages can give a more comprehensive comprehension of the spatiotemporal heterogeneity of Tregs.

Further advancement in the development of immune checkpoints for Tregs and their inhibitors necessitates more precise definitions of subpopulations. The variability in the expression of immunosuppressive markers CTLA4, TIM3, TIGIT, and LAG-3 on Tregs indicates the existence of possible subsets with distinct functional properties. During the development of the immunosuppressive microenvironment surrounding tumors, various subpopulations of Tregs expressing diverse immunosuppressive molecules are crucial, each having distinct immune checkpoints [168, 169]. Multiple PD-1/PD-L1 and CTLA-4 inhibitors have been extensively employed in clinical settings, prolonging survival rates among cancer patients by suppressing Tregs activities. However, their efficacy is not always guaranteed [170]. Low CTLA-4 and PD-1 expression in Tregs from glioma-bearing mice suggests that other immune checkpoints may play an important role in immunosuppression in the glioma microenvironment [171]. Currently, the development of immune checkpoint therapy is shifting focus from CTLA-4 and PD-1 to TIGIT and TIM-3. In TME, certain Tregs express both TIGIT and TIM-3. Monoclonal antibodies directed at TIGIT and TIM-3 can decrease the repressive impact of TIM-3^+^TIGIT^+^ Tregs cells on CD8^+^ T cells and augment the killing of tumor cells [71]. Several anti-TIGIT drugs are currently in clinical development, with the most rapidly advancing ones being tiragolumab, ociperlimab, and domvanalimab, all of which are in clinical phase III. Combining multiple immune checkpoint inhibitors can target various pathways and potentially enhance efficiency.

Tregs inhibit the systemic inflammatory response caused by excessive activation of anti-tumor immunity, play an indispensable role in the maintenance of immune homeostasis, and prevent and ameliorate tissue cells damage induced by immunotherapy [172]. The immune system recognizes and destroys tumor cells. Following tumor cells death, specifically immunogenic death, more tumor-associated antigens are released. This triggers the activation of additional T cells to target and eliminate the tumor [173]. When over-activated, this anti-tumor immune response cross-reacts with the body's own tissue cells, ultimately resulting in autoimmune disease and systemic inflammation. These outcomes have been found to have a poor prognosis [174]. Chimeric antigen receptor T-cell (CAR-T) therapy is a promising new form of cellular immunotherapy. It has demonstrated efficacy in treating hematologic and solid tumors. However, its clinical application is constrained by the frequent occurrence of cytokine release syndrome (CRS) as an adverse event [175]. Elevated levels of C-reactive protein (CRP), an acute-phase protein, could signify an unfavorable pro-inflammatory immune response, which measures systemic inflammatory response syndrome (SIRS) and CRS in a range of cancers associated with poorer prognosis [176]. Nonsteroidal anti-inflammatory drugs (NSAIDs), a type of immunosuppressive drug that affects the whole body, block cyclooxygenase-2 (COX-2) and therefore decrease the presence of inflammatory factors. NSAIDS are utilized as adjunctive treatment for tumors displaying elevated CRP values [177]. Tregs were shown to down-regulate COX-2 expression and produce anti-inflammatory effects in an assay using mouse intestinal adenomas as a model [117]. Additionally, Tregs exhibit more targeted and secure immunosuppression than NSAIDs in vivo, mainly by curbing the activation and operation of immune cells [178]. Supplemental Tregs modulate adverse effects of anti-tumor immunity and improve prognosis in patients with tumors [179]. Tregs supplementation can modulate the adverse effects of anti-tumor immunity and contribute to improved outcomes in tumor patients. Therefore, in the future, exploring and monitoring a moderate density interval of Tregs during anti-tumor immunotherapy should be considered to improve anti-tumor efficacy without harming the normal tissues of patients.

**Fig. 4:**
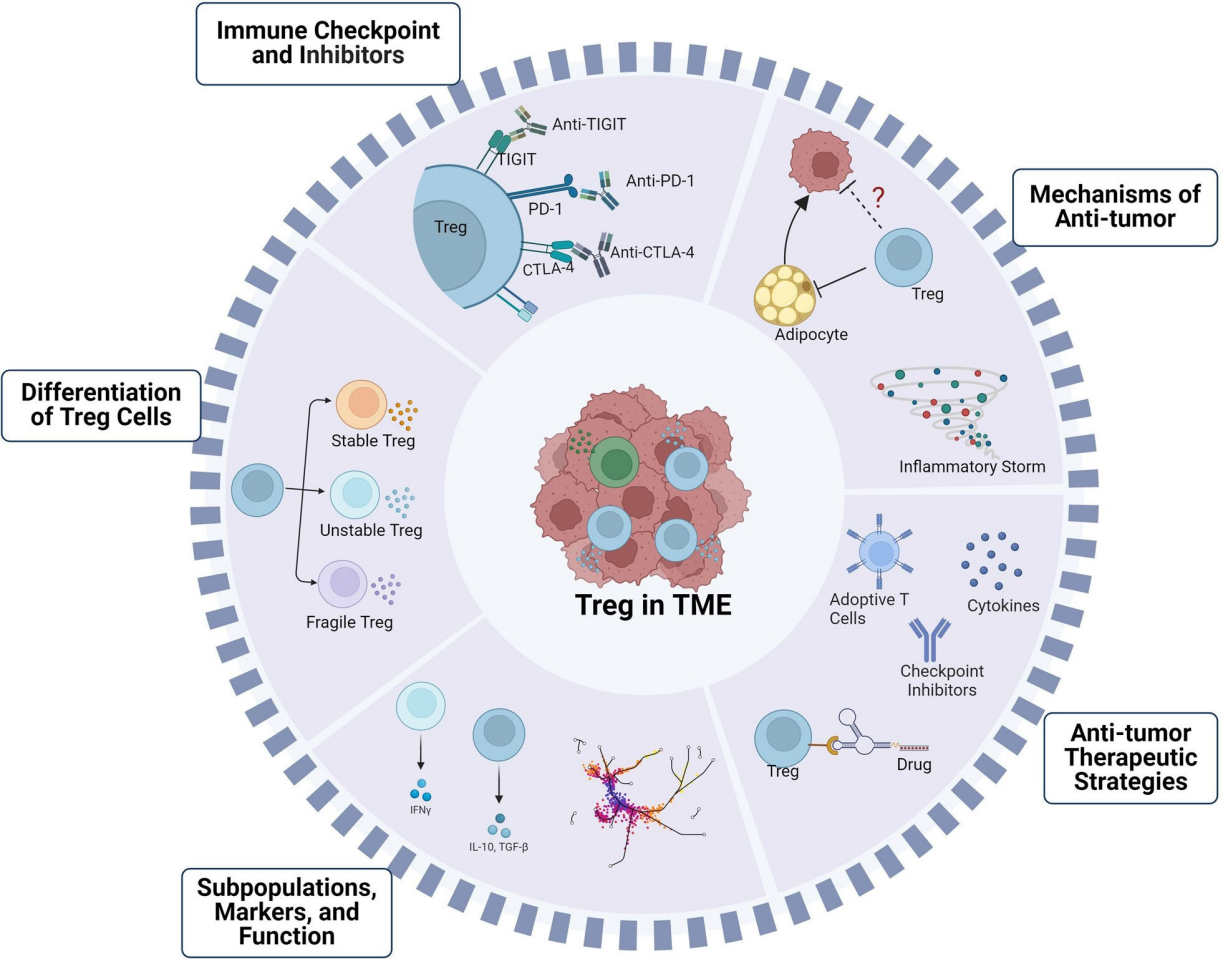
Future perspectives of Tregs in TME

The potential anti-tumor effects of Tregs require further investigation and verification. Tregs have both positive and negative impacts on the tumorigenesis and progression of tumors. On the one hand, Tregs suppress the death of tumor cells by lowering the anti-tumor immune response in late-stage tumors. In the early stages of tumors, Tregs exert immunosuppressive effects that suppress inflammation in favor of tumor progression, for example, Tregs have the ability to inhibit Th17-mediated inflammatory anti-microbial response in both the tumor and the gut. Several types of Tregs have been identified in the intestinal mucosa, including RORγT^+^Helios^−^ Tregs induced by gut microbes and GATA3^+^ Helios^+^ Tregs originating from the thymus. These Tregs respond to tissue damage-induced IL-33 and have the capability to restrict tissue damage and prevent tumorigenesis during colitis [25, 180–182]. Certain subpopulations of Tregs have the ability to promote tumor cells killing, either through phenotypic conversion or functional specialization. The anti-tumor mechanisms of Tregs discussed in this article require further experimental data to be validated. Furthermore, there are numerous areas meriting additional investigation in this avenue. Could Tregs have an indirect impact on tumors via interaction with adipocytes? Excessive adipocyte accumulation in the body incites cellular stress, including endoplasmic reticulum and oxidative stress, eventually culminating in a chronic inflammatory microenvironment [183, 184]. This pro-inflammatory environment heightens the risk of tumorigenesis through hormonal alteration and releases numerous cytokines and adipokines (such as IL-6, TNF-a, leptin, and adiponectin), which trigger various tumor-associated signal pathways [185]. For instance, leptin can induce epithelial-mesenchymal transition (EMT) by binding to leptin receptor (Ob-R) and enhance tumor migration through interaction with Rho family GTPases [186]. Free fatty acids, ketone bodies, and amino acids produced by adipocytes can provide energy for the proliferation and metastasis of tumors [187]. Tregs display high expression of peroxisome proliferator-activated receptor gamma (PPARγ) in visceral adipose tissue (VAT) and exert an inhibitory effect on adipose tissue inflammation [188]. Furthermore, a study conducted by transferring Tregs transversely to an obese mouse model showed a noteworthy decline in visceral adipocyte diameters in comparison to the control group [189]. Inhibiting Tregs in the chronic inflammatory microenvironment induced by adipocytes may suppress tumors. However, this hypothesis requires further experimental verification (Fig. 4).

## Conclusion

Since the discovery of Tregs as vital mediators of autoimmune tolerance, their involvement in tumors has frequently been viewed as adverse. Recent studies have revealed the existence of subpopulations of Tregs with different phenotypes and functions within tumors, and have led to the development of new directions for targeted therapies. We have summarized that Tregs can inhibit tumorigenesis and progression by enhancing anti-tumor immunity and suppressing inflammation that promotes tumor progression, which has led to a revision of the current one-sided understanding of Tregs in the TME. Interestingly, we have further illustrated that the role of Tregs in TME can be modified based on the tumor's type, location, and stage, which supports future refinement of anti-tumor strategies for Tregs. However, the majority of the studies analyzed in this section focused solely on clinical data. It is crucial to conduct further investigations into the biological basis of Tregs, which hinders the advancement of various cancer types and influences diverse clinical outcomes. In addition, further research is needed to determine how to use Tregs as prognostic factors and therapeutic strategies for different tumors due to the complexity of TME and the diversity of Tregs’ functions.

## Data Availability

Not applicable.

## Abbreviations

TME: Tumor microenvironment
Tregs: Regulatory T cells
FoxP3: Forkhead box protein 3
CTLA-4: Cytotoxic T lymphocyte-associated antigen-4
PD-1: Programmed death receptor 1
ICOS: Inducible T-cell costimulator
GITR: Glucocorticoid-induced TNFR-related protein
TIGIT: T cell immunoglobulin and ITIM domain
LAG-3: Lymphocyte activation gene 3
Nrp1: Neuropilin-1
Teffs: Effector T cells
CNS2: Conserved non-coding sequence 2
TSDR: Tregs-specific demethylation region
TNFR: Tumor necrosis factor receptor
Sema4A: Semaphorin 4A
BATF: Basic leucine zipper ATF-like transcription factor
IDO: Indoleamine 2,3-dioxygenase
TGF-β: Transforming growth factor-β
IFN-γ/IFN-γR: Interferon-gamma/interferon-gamma receptor
AML1: Acute myeloid leukemia-1
ICI: Immune checkpoint inhibitor
GATA3: Gata binding protein 3
CTL: Cytotoxic T lymphocytes
TCR: T-cell receptor
MHC: The major histocompatibility complex
OX-40: Tumor necrosis factor receptor superfamily, member 4
FcεRI: FcepsilonRI
LPS: Lipopolysaccharide
GzmB: Granzyme B
M2φ: M2 like macrophage
Stat5: Signal transducer and activator of transcription 5
VEGF: Vascular endothelial growth factor
myCAFs: Cancer-associated myofibroblast
CCL3/6/8: The chemokine C–C motif chemokine ligand 3/6/8
CCR4: The CC chemokine receptor 4
Arg1 + Mφ: Arginase 1 positive macrophage
MRs: Master regulators
ETBF: Enterotoxigenic bacteroides fragilis
CRP: C-reactive protein
G-CSF: Granulocyte colony-stimulating factor
CAR-T: Chimeric antigen receptor T-cell
CRS: Cytokine release syndrome
iRAEs: Immune-related adverse events
SIRS: Systemic inflammatory response syndrome
CBM: CARD9-BCL10-MALT1
EMT: Epithelial-mesenchymal transition
Ob-R: Leptin receptor
PPARγ: Peroxisome proliferator-activated receptor gamma
VAT: Visceral adipose tissue
